# Transforming Primary Care Data Into the Observational Medical Outcomes Partnership Common Data Model: Development and Usability Study

**DOI:** 10.2196/49542

**Published:** 2024-08-13

**Authors:** Mathilde Fruchart, Paul Quindroit, Chloé Jacquemont, Jean-Baptiste Beuscart, Matthieu Calafiore, Antoine Lamer

**Affiliations:** 1 Univ Lille, CHU Lille, ULR 2694 - METRICS: Évaluation des Technologies de santé et des, Pratiques médicales, 2 Place de Verdun, Lille, F-59000, France; 2 Département de Médecine Générale, University of Lille, Lille, France; 3 F2RSM Psy - Fédération régionale de recherche en psychiatrie et santé mentale Hauts-de-France, Saint-André-Lez-Lille, France

**Keywords:** data reuse, Observational Medical Outcomes Partnership, common data model, data warehouse, reproducible research, primary care, dashboard, electronic health record, patient tracking system, patient monitoring, EHR, primary care data

## Abstract

**Background:**

Patient-monitoring software generates a large amount of data that can be reused for clinical audits and scientific research. The Observational Health Data Sciences and Informatics (OHDSI) consortium developed the Observational Medical Outcomes Partnership (OMOP) Common Data Model (CDM) to standardize electronic health record data and promote large-scale observational and longitudinal research.

**Objective:**

This study aimed to transform primary care data into the OMOP CDM format.

**Methods:**

We extracted primary care data from electronic health records at a multidisciplinary health center in Wattrelos, France. We performed structural mapping between the design of our local primary care database and the OMOP CDM tables and fields. Local French vocabularies concepts were mapped to OHDSI standard vocabularies. To validate the implementation of primary care data into the OMOP CDM format, we applied a set of queries. A practical application was achieved through the development of a dashboard.

**Results:**

Data from 18,395 patients were implemented into the OMOP CDM, corresponding to 592,226 consultations over a period of 20 years. A total of 18 OMOP CDM tables were implemented. A total of 17 local vocabularies were identified as being related to primary care and corresponded to patient characteristics (sex, location, year of birth, and race), units of measurement, biometric measures, laboratory test results, medical histories, and drug prescriptions. During semantic mapping, 10,221 primary care concepts were mapped to standard OHDSI concepts. Five queries were used to validate the OMOP CDM by comparing the results obtained after the completion of the transformations with the results obtained in the source software. Lastly, a prototype dashboard was developed to visualize the activity of the health center, the laboratory test results, and the drug prescription data.

**Conclusions:**

Primary care data from a French health care facility have been implemented into the OMOP CDM format. Data concerning demographics, units, measurements, and primary care consultation steps were already available in OHDSI vocabularies. Laboratory test results and drug prescription data were mapped to available vocabularies and structured in the final model. A dashboard application provided health care professionals with feedback on their practice.

## Introduction

The digitalization of health care organizations has made it possible to automatically collect and reuse data from electronic health records (EHRs) for care, administrative, and research purposes [1]. Data reuse generally relies on extracting data from source databases, formatting and normalizing it in a data warehouse [2-5]. Over the last few years, hospital-based data warehouses have started to provide comprehensive overviews of patient management during a hospital stay or on a hospital ward. However, these data warehouses do not contain data on primary care or other data not related to the hospital stay. These data cover first-line services—outpatient care provided in local practices, including general practice, community pharmacy, dental care, and optometry [6,7]. Additionally, data from all individuals covered by the French national health insurance scheme are anonymously and prospectively included in the national claims database [8]. These data are used for reimbursement purposes and are not clinical.

The reuse of EHR data is now a major topic of interest for hospital care [9,10] and primary care [4,11]. Several research groups have retrospectively reused primary care data on patients with neuromuscular diseases [12], diabetes [4,13], dermatological diseases [14,15], lung diseases [16], cancer [17], or urinary tract infections [18] or on older adult patients [19]. Several national projects aim to collect primary care data on a routine basis. The main primary care projects are the Clinical Practice Research Datalink and The Health Improvement Network in the United Kingdom [20,21], the Veterans Administration data warehouses in the United States [22], and the Canadian Primary Care Sentinel Surveillance Network in Canada [23]. Nevertheless, the reuse of health care data (and especially primary care data) faces many challenges [9,10,24-27]. The abovementioned projects collect data from millions of patients by implementing local data models. A UK project uses a national database (BioBank) to standardize vaccination data into a common data model (CDM) format [28].

The heterogeneous structure of the data and the use of country- and facility-specific vocabularies create barriers to the implementation of multicenter studies and the sharing of data, methods, and results. Initiatives such as those proposed by the Observational Health Data Sciences and Informatics (OHDSI) consortium seek to (1) standardize data structure and vocabularies; (2) promote reproducible research and collaboration; and (3) share methods, tools, and results [29,30]. The OHDSI has notably developed the Observational Medical Outcomes Partnership (OMOP) CDM [31] and provides standard data structures and vocabularies that are independent of individual software developers and countries [32].

Hospital and claims databases have already undergone the mapping process to adopt the OMOP CDM format [33-35]. Nevertheless, primary care data, specifically general practice data, which serve as a valuable addition to hospital and claims databases, are still rarely being integrated into the OMOP CDM format.

Data standardization might facilitate the development of common tools for health care professionals, such as activity dashboards and software for managing multicenter research projects. Hence, the primary objective of this study (part of the Primary Care Data Warehouse [PriCaDa] project) was to transform primary care data into the OMOP CDM format.

## Methods

### Overview

The goal of the PriCaDa project is to reuse primary care data and to provide an overview of the practices of health care professionals working in primary care (family physicians, nurses, and pharmacists).

**Figure 1.**
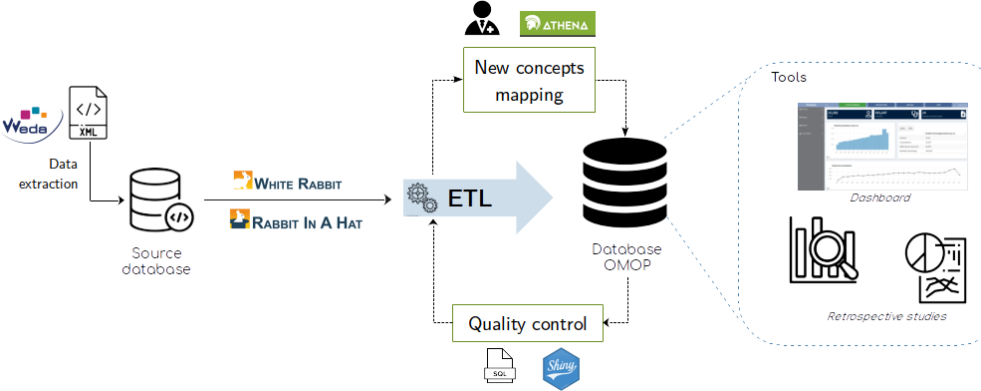
Pipeline of the primary care data transformation into the OMOP CDM. CDM: Common Data Model; ETL: extract-transform-load; OMOP: Observational Medical Outcomes Partnership.

In this study, we used primary care data from a multidisciplinary health center (MHC) located in the town of Wattrelos, France, in collaboration with the software company that produced the MHC’s EHR software (Weda). Weda ranks third in terms of sales volume, boasting over 20,000 health care professionals in France as users [36]. We used an extract-transform-load (ETL) process to create a data warehouse (Figure 1), using Python and SQL scripts. The data warehouse was stored in a PostgreSQL database, using version 5.4 of the OMOP CDM [31]. The first step in the ETL process was to extract, classify, and normalize entities from the XML files. The database was then scanned and assessed with the OHDSI tool White Rabbit [37] to create a report on each raw table, attribute, and data type. The OHDSI tool Rabbit In A Hat was then used to implement the specifications of the structural mapping, which aims to associate each local table and local column to the corresponding OMOP nomenclature [38]. Then, transformation steps normalize the structure and the semantic of the data source to the OMOP CDM format. The final step is to load the data into the OMOP CDM. The development of a dashboard was proposed for a practical application.

### Ethical Considerations

The study was approved by the staff at the MHC in Wattrelos and the management of the Weda software house. Furthermore, the study was registered with the French National Data Protection Commission (*Commission nationale de l’informatique et des libertés*; reference 2022‐203). In line with the French legislation on retrospective studies of anonymized data from routine clinical practice, approval by an institutional review board was not required [39]. All the data collection and analysis methods complied with relevant guidelines and regulations. Patient confidentiality was maintained at all times, and all the data were anonymized before extraction. The Weda software exports data with an identifier for each patient and consultation. This identifier is replaced during the transformations with a unique artificial ID. For this unique ID, sequences provided by the database management system (ie, PostgreSQL) were generally used to create an autoincrement primary key.

### Data Extraction

Following approval by the MHC’s staff, Weda exported an XML-based hierarchical structure file for each patient. A primary care consultation occurs in 4 steps. At each step, the family physician collects data on the patient’s health status. In step one (arrival), the reason for consultation is recorded as free text. In step two (the interview), the physician records clinical signs and symptoms as free text. In step three (the examination), the physician makes biometric measurements and enters the data in a structured way in the appropriate fields of the software (variables, values, and units). Step four (the outcome) includes the diagnosis (if made, as free text), any drug prescriptions (documented with the *Code Identifiant de Spécialité* [CIP; the French national drug code]), any referrals (as free text in a PDF letter), and information on vaccinations. The parts of the Weda software dedicated to these steps depend on the physician’s habits; some physicians put all observations into a single field.

Furthermore, the family physician receives laboratory test results from medical laboratories and clinical reports from other specialist physicians. The test variables differ from one medical laboratory to another. For example, one laboratory might use the term “creatinine” and a specific unit, whereas another might use “create” and a different unit for the same variable. These reports were documented as free text.

### Transformation Steps

#### Semantic Mapping

The goal of semantic mapping was to link local vocabularies to standard vocabularies that have been defined by the OHDSI community and are available in an web-based tool [32,40]. The difficulty of the mapping depends on the vocabularies available in the source data. We defined four difficulty levels: level 1 for local vocabulary items that already belonged to the standard OHDSI vocabulary; level 2 for local, nonstandard vocabulary items for which the mappings to the standard OHDSI vocabulary already existed; level 3 for local vocabulary items that did not correspond to the OHDSI vocabulary but were in a structured format (manual mapping was necessary); and level 4 for local vocabulary items that did not correspond to the OHDSI vocabulary and were in an unstructured format (free text). Natural language processing (NLP) techniques, such as fuzzy matching algorithms, SpaCy tools, and regular expressions, can be used for concept identification and information extraction. The free text (eg, reasons for consultation, symptoms, diagnosis, and clinical reports) was sometimes heterogeneous and unstructured. Indeed, a single free-text record can consolidate various types of information, including the reason for a consultation, the patient’s clinical signs, and their diagnosis, all of which may vary based on the practices of the individual family physician.

For difficulty levels 2 and 3, the data were mapped manually and independently by two experts (MF and CJ). A κ score was computed as a statistical metric for assessing the degree of consensus among the annotators tasked with assigning labels to data. It gauges the degree to which the annotators’ assessments align, while also considering the potential for chance agreement. The score ranges from 0 to 1 (1 for perfect agreement among annotators). A κ score below 0.4 indicates weak agreement, a κ score above 0.6 (60%) suggests moderate agreement, and a κ score exceeding 0.8 (80%) signifies strong agreement [41]. Any disagreements were settled by a third expert (PQ). This mapping provided correspondences between the local vocabulary items and standard vocabulary items. When necessary, new primary care concepts not available in the OHDSI vocabularies were loaded into the CONCEPT table. The Logical Observation Identifiers Names and Codes vocabulary was used to map laboratory test variables. The source laboratory test concepts contained the name of the laboratory test variable used by the laboratory, together with the unit. The text referencing the laboratory test variables was cleaned up by grouping equivalent source concepts. Punctuation and special characters were removed, and abbreviations or spelling differences were replaced and grouped under the full name (eg “CRP” was replaced with “C-reactive protein”). Chapter numbers or line numbers at the beginning of a line were removed. Multiple spaces were replaced by a single space, and stop words (eg, “of,” “to,” or “an”) were removed. The Systematized Nomenclature of Medicine was used to map biometric variables, with the Unified Code for Units of Measure for units of measurement and the *International Classification of Diseases, 10th Revision* (*ICD-10*) for the patient’s medical history. Drug prescriptions were recorded using CIP drug name codes. The CIP code was extracted from the Weda EHR software and mapped to the Anatomical Therapeutic Chemical (ATC) code and then the RxNorm code (ATC to RxNorm mapping is already available in the OMOP CDM).

The new concepts were integrated into the CONCEPT table with a concept identifier greater than 2,000,000,000. When a local concept is mapped, the correspondence with a standard OMOP concept is loaded into the CONCEPT_RELATIONSHIP table (resulting in a link between a local concept identifier and a OMOP standard concept identifier). As a result, the identifiers of standard concepts can be loaded into the x_concept_id column of the corresponding table for the local concept (eg, new local concepts in the MEASUREMENT table that are mapped are associated with the standard concepts loaded into the measurement_concept_id column).

#### Structural Mapping

The goal of structural mapping was to transform the source structure into the OMOP CDM structure. The mapping comprised two steps. In the first step, the relevant variables required for the OMOP model were selected and normalized in a table format. In the second step, the variables and table structures were transformed to match the OMOP CDM nomenclature. We observed that most of the medical histories were coded as free text, rather than nomenclature items. The DIAGNOSIS source table contained the information from the patient interview (clinical signs), the clinical examination (measurements), and the “outcome” parts of the consultation. However, this field contained data related to several types of medical information in text format. The MEASUREMENT table contained information from the “laboratory data” and “biometrics” source tables. Outliers were removed, and primary and foreign keys were identified. New artificial identifiers were created for the primary key in each table.

### Data Loading and Quality Assessment

After the semantic and structural transformations, the data were loaded into the OMOP model. We used Achilles, a data quality assessment and visualization tool developed by the OHDSI community. Achilles reports the compliance of the mapping with the constraints of the OMOP CDM (the primary and foreign keys), the vocabulary (ie, the correct choice of concepts corresponding to each table), and the business rules (ie, rules that ensure data consistency, for example, data chronology respects real life). Based on the Achilles analysis tables, the Atlas server summarizes the results of the data quality assessment in a dashboard [42]. Kahn et al [43] have developed a data quality framework for the secondary use of EHR data integrated into the Atlas quality assessment dashboard. *Conformance* describes “the compliance of the representation of data against internal or external formatting, relational, or computational definitions.” *Completeness* computes “features that describe the frequencies of data attributes present in a data set without reference to data values.” *Plausibility* describes “the believability or truthfulness of data values.” The data quality assessment context *verification* is a strategy “for the source of expectations or comparisons of EHR data based on internal characteristics” [43].

To test the relevance and usability of primary care data in the OMOP CDM, we compared the results of several queries of the OMOP CDM with the results obtained directly from the Weda software. The queries were run by a physician (CJ) who used the software in his clinical practice. To ensure that the patient records could be checked manually, the queries were chosen to keep the resulting number of patients low. Two queries corresponded to the MHC’s general activity (eg, the number of patients per family physician), two corresponded to prescription data, and a fifth query corresponded to laboratory test data.

### Practical Application

Using RShiny and the *shiny*, *shinyBS*, *shinycssloaders*, *shinydashboard*, *shinyjs,* and *shinyWidgets* libraries, we implemented a dashboard to report the MHC’s general activity, prescriptions, and the distribution of the laboratory test results [44].

## Results

### Data Extraction

The data were extracted in July 2021. The available patient profiles dated back to 1997, and data on clinical measurements, drug prescriptions, and laboratory tests were available from 2013 onward. The extracted data contained 18,395 patient files. Each patient’s file contained anonymized demographic information (ie, sex, year of birth, the town or city of residence, and the country of residence), the date of the first consultation, and the name of the family physician with whom the patient was registered. It also contained the patient’s medical history (documented with *ICD-10* codes or as free text), information related to the consultation, laboratory test results, and clinical reports from other physicians (also as free text).

### Transformation

#### Semantic Mapping

In all, 17 vocabularies were mapped (Table 1).

All the new concepts were added to the CONCEPT table (n=10,221). These primary care concepts were added alongside the existing concepts in the OMOP model developed by OHDSI. The mapping between local concepts and standard concepts was integrated into the CONCEPT_RELATIONSHIP table (n=9432).

**Table 1.** The concept mapping. When a local vocabulary is not given, it means that the concept had to be created.

Feature	Local vocabulary	OMOP^a^ vocabulary	Level of mapping difficulty	Concepts, n	Mapped concepts, n/N (%)	Associated records, n/N (%)
Care site	—^b^	Care site	Level 1	1	1/1 (100)	1/1 (100)
Medical histories	*ICD-10* ^c^	*ICD-10*	Level 1	83	80/83 (96.4)	2252/2315 (97.3)
Visit	—	Visit	Level 1	2	2/2 (100)	592,226/592,226 (100)
Drug	ATC^d^	RxNorm	Level 2	9070	9100/9100 (100)	684,805/684,805 (100)
Drug	CIP^e^ code	ATC	Level 3	9946	9100/9946 (91.5)	684,805/814,772 (84)
Biometric variables	Free text in structured fields	SNOMED^f^	Level 3	243	12/243 (4.9)	172,549/179,337 (96.2)
Laboratory test variables	Free text in structured fields	LOINC^g^	Level 3	2312	170/2312 (7.4)	829,498/941,522 (88.1)
Measurement units	Free text in structured fields	UCUM^h^	Level 3	217	65/217 (30)	863,259/1,120,859 (77)^i^
Patient characteristics	Free text in structured fields	Sex	Level 3	2	2/2 (100)	18,395/18,395 (100)
Outcome (diagnosis)	Free text	—	Level 4	—	—	—
Consultation (reason)	Free text	—	Level 4	—	—	—
Examination (measure taken)	Free text	—	Level 4	—	—	—
Clinical report by another physician	Free text	—	Level 4	—	—	—
Medical history	Free text	—	Level 4	—	—	—
Referrals	Free text	—	Level 4	—	—	—
Supplementary information	Free text	—	Level 4	—	—	—
Vaccine prescription	Free text	—	Level 4	—	—	—
Vaccination	Free text	—	Level 4	—	—	—

a OMOP: Observational Medical Outcomes Partnership.

b Not applicable.

c *ICD-10: International Classification of Diseases, 10th Revision.*

d ATC: Anatomical Therapeutic Chemical.

e CIP: *Code Identifiant de Spécialité* (the French national drug code).

f SNOMED: Systematized Nomenclature of Medicine.

g LOINC: Logical Observation Identifiers Names and Codes.

h UCUM: Unified Code for Units of Measure.

i 23% (257,801/1,120,859) not available.

More than 80% (9100/9946, 91.5%) of the drug records and more than 90% (80/83, 96.4%) of the *ICD-10*–coded medical history records were mapped. Less than 4.9% (12/243) of the biometric measurement concepts were mapped; the latter accounted for 96.2% (172,549/179,337) of the records because a small number of concepts were used (eg, weight, height, and heart rate; Table 1). The remaining records were free-text and family physician–dependent variables. The writing style can vary and the same variable can be asked in several ways. For example, to find out whether the patient is a smoker, the family physician can input the information in the family physician–dependent variable “does my patient smoke?” “is he a smoker?” or “do you smoke?”

For level 3 mapping difficulties, drug-related concepts (coded as CIP codes) were mapped in two steps. RxNorm is the standard classification chosen by OHDSI for the OMOP model, and therefore, we had to map our local terminology (ie, CIP) to RxNorm. First, the CIP codes were mapped to the ATC codes. Second, the ATC codes were mapped to the RxNorm codes, using the correspondences already implemented in the CONCEPT_RELATIONSHIP table.

With regard to the laboratory test variables, the cleaning step reduced the number of concepts from 3003 to 2312. We restricted the mapping to the most frequently cited laboratory test concepts in the MEASUREMENT table, with the aim of covering more than 80% of the records. Disagreements over laboratory test concept mapping by experts were resolved by consensus and the involvement of a third annotator. The experts disagreed about 24.3% (37/152) of the mapped concepts, which corresponds to a κ score of 75%.

#### Structural Mapping

**Figure 2.**
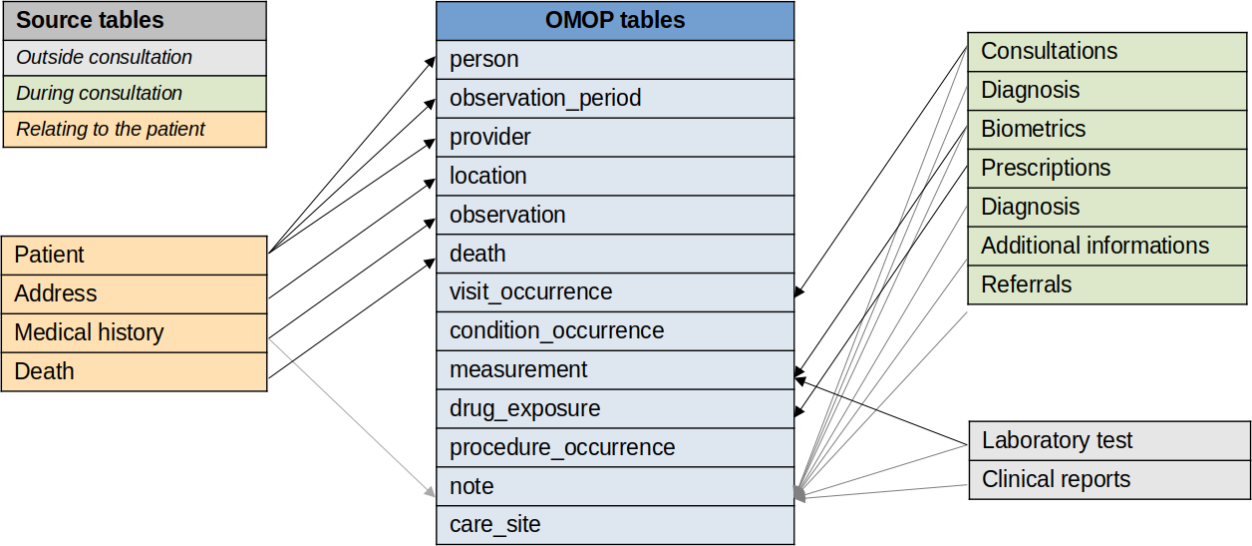
Structural mapping of each source variable to the OMOP CDM format. Common Data Model; OMOP: Observational Medical Outcomes Partnership.

Several XML tables in the source model corresponded to tables in the OMOP CDM format (Figure 2). Information about the patient, the family physician, the profile creation date, and the health care center was stored in the PERSON, PROVIDER, OBSERVATION_PERIOD, and CARE_SITE tables, respectively. The medical history (documented with *ICD-10* codes) was stored in the OBSERVATION table. Each consultation at the health care center corresponded to a record in the VISIT_OCCURRENCE table, identified by a visit_concept_id (A*mbulatory Primary Care Clinic/Center*; concept_id=38004247). Biometric measurements were stored in the MEASUREMENT table, with a concept type corresponding to *EHR physical examination*. Laboratory test results were stored in the MEASUREMENT table, with a concept type *Lab* (concept_id=32,856). The feature measurement_source_type_id distinguishes each source table (laboratory data or biometrics measurements). Each drug prescription was stored in the DRUG_EXPOSURE table. Free-text information collected during the consultation was stored in the NOTE table with an appropriate source vocabulary concept identifier (eg, a note of clinical signs; the reason for the consultation; medical histories; the outcome of the consultation; and, in some cases, the associated diagnosis). Medical reports from a specialist physician not based in the MHC were recorded in the NOTE table (Figure 2).

All the standard concept types used to identify information in the various source tables are detailed in Multimedia Appendix 1; for example, the NOTE table contains information about the reason for the consultation, the patient’s medical history, and other medical reports.

### Data Loading and Quality Assessments

Records spanning 20 years (592,226 consultations by 18,395 patients) were integrated into the OMOP CDM. The numbers of records per OMOP table before and after the ETL process and the related computing time are reported (Table 2). The CARE_SITE and DRUG_ERA tables were implemented using data transformed into the OMOP format.

The Atlas Server dashboard produced the data distributions for each table. An example of the distribution of blood potassium concentrations is shown in Multimedia Appendix 2.

**Figure 3.**
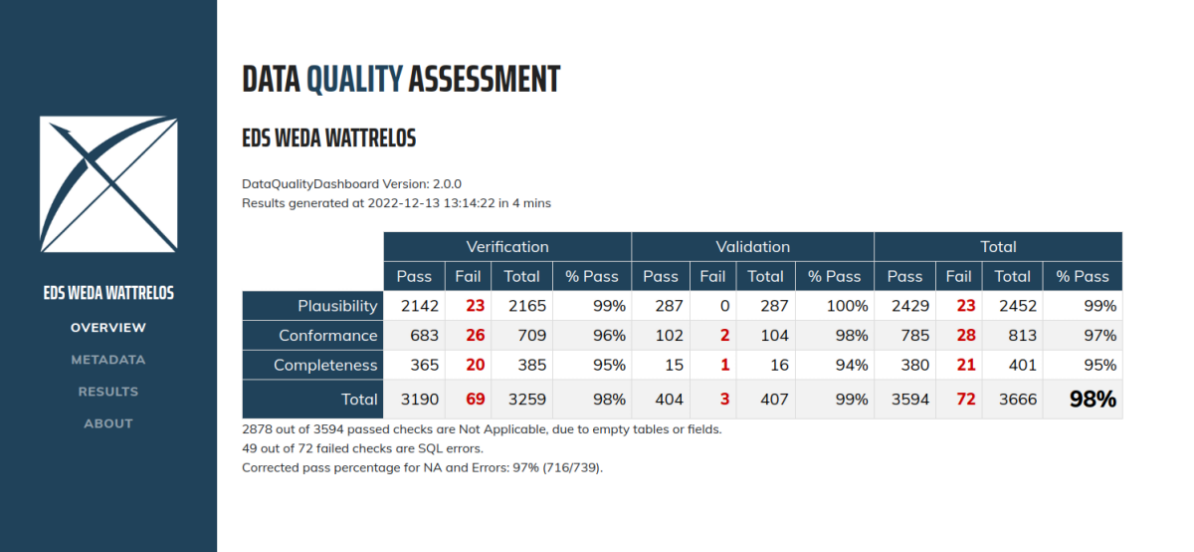
The data quality assessment dashboard. NA: not available.

The Atlas dashboard’s overview tab provided a top-level summary of the total number of passes and failures by Kahn category (Figure 3). We found 28 *Conformance* failures that did not respect the specifications of the OMOP CDM. There were 21 *Completeness* failures related to potentially missing data and 23 failures related to *Plausibility* for implausible dates or measurement values. For each of these failures, the results tab (Multimedia Appendix 3) displayed one line per *verification*.

**Table 2.** The volume of each table before and after the ETL^a^ process and the associated computing time.

OMOP^b^ tables	Records before the ETL process, n	Records after ETL process, n	Computing time (s)	Local tables
CARE_SITE	—^c^	1	<0.001	—
DEATH	419	419	<0.001	Death
DRUG_ERA	—	1,084,012	3.75	—
DRUG_EXPOSURE	924,216	814,772	4.54	Drug
LOCATION	19,662	11,433	0.01	Address
MEASUREMENT	1,120,859	1,120,859	225.76	Biometrics and laboratory test
NOTE	2,772,809	2,091,705	4.57	Referrals, consultations, diagnoses, clinical reports from outside the MHC^d^, medical history, additional information, and vaccination status
OBSERVATION	64,669	2315	0.16	Medical history
OBSERVATION_PERIOD	—	18,256	0.01	—
PERSON	18,395	18,395	0.04	Patient
PROVIDER	8	8	—	Patient
VISIT_OCCURRENCE	592,227	592,226	1.54	Consultation

a ETL: extract-transform-load.

b OMOP: Observational Medical Outcomes Partnership.

c Not applicable.

d MHC: multidisciplinary health center.

The concordance of results obtained from data quality evaluation queries between the source EHRs and the data warehouse’s records proved that the process was reliable (Multimedia Appendix 4). In the event of a difference between the two (results obtained from the data warehouse in OMOP format and results from the Weda software), we had to check the patient records in the software to identify what the correct figure was. Patients whose profile had been created after 2021 had to be removed from the results produced by the Weda software because the extraction data (stored in the data warehouse) goes up to June 2021 at the latest, and queries on the software interface allow filtering for the entire year. The family physician must declare when he or she became the patient’s referring family physician. This family physician declaration is dated in the Weda software. This date is not found in the data warehouse. The date of the patient’s registration with the family physician was also taken into account in the queries; registrations after the extraction date were removed. Queries concerning the laboratory test results were checked manually by one of the MHC’s family physicians (CJ). Some laboratory test results were saved in the “reports” part and others were saved in the “consultations” part on the software.

### Practical Application

**Figure 4.**
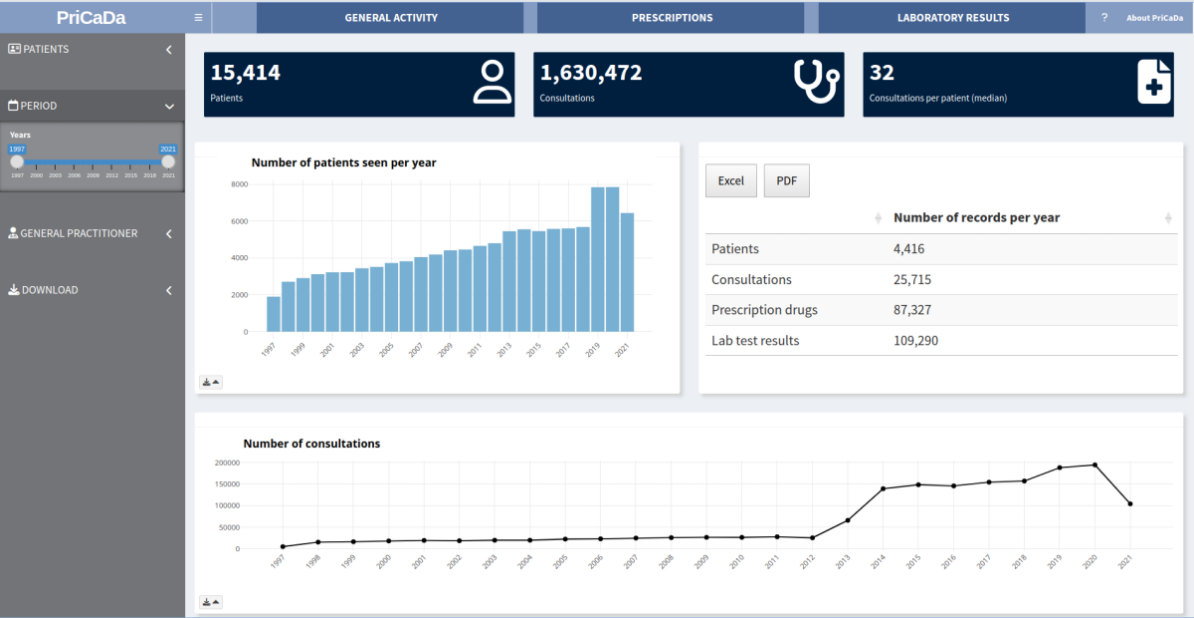
The prototype primary care dashboard. PriCaDa: Primary Care Data Warehouse.

A 3-tab prototype dashboard was implemented (Figure 4). The first tab concerned the MHC’s activity, the second concerned drug prescriptions, and the third concerned laboratory test results.

## Discussion

### Principal Findings

In this study, we implemented primary care data into the OMOP CDM format from a French health care facility. Five concepts related to the consultation (reason for the consultation, diagnosis, and comments), the clinical report by another physician, some medical histories, referrals, and vaccination data in free text were stored in the NOTE table, and three structured free-text concepts were mapped to standard concepts (laboratory test results, biometrics, and measurement units). Overall, we included 592,226 consultations and 10,221 concepts over 20 years, including 9432 mappings between local concepts and standard concepts. The concept mapping was validated by three experts, including a family physician. We then used queries to validated the design of the OMOP CDM by comparing the results obtained in our data warehouse with those obtained in the source software.

The OMOP model was originally developed to answer pharmaco-epidemiologic questions by reference to hospital databases or claims databases. New types of data have since been integrated, such as those in the fields of cancer, microbiology, and anesthesia [32,33,45]. Integrating these new data types might present difficulties not foreseen in the original CDM. Integrating primary care data required the addition of 10,221 new concepts. Through the sharing of tools and methods, the OMOP model enables reproducible analyses of decentralized data [28,46].

### Comparison With Previous Work

This work stands out for its integration of out-of-hospital clinical data over a long time period. In contrast to the French national health care database (*Système National des Données de Santé*), we included clinical data. Similarly, the data produced by French hospital included test results from the hospital’s laboratory only. We were able to load data from outpatient treatments and out-of-hospital medical laboratories into the OMOP model; in France, these data are not documented in claims and hospital databases. We also have data on drug prescriptions and associated dosage (duration of treatment, number of refills, number of drugs per dose, and dosing period), whereas the *Système National des Données de Santé* only contains data on prescription fulfillment.

By using a CDM, we will be able to share our work with other primary care data reuse initiatives [28].

This study had a number of strengths. First, it was based on collaboration between data scientists and family physicians. Each data transformation step was approved by the MHC’s family physicians. Second, working with the EHR software’s developer enabled us to understand the software’s structure and export format. The involvement of the software developer in the study expedited the data extraction process. This collaboration with the software developer allowed us to retrieve the data in XML format, comprehend each of the XML tags, and discern the origin of the XML information within the software. Third, our development of a dashboard gave health care professionals an overview of their practice in terms of the number of patients followed and the number of consultations carried out over a defined period.

### Limitations

The first limitation is that a large proportion of the extracted primary care data had been entered as free text and required NLP methods or manual examination to be used secondarily. Moreover, the primary care EHR software provides information on drug prescriptions but not on filling or patient compliance.

The free text was difficult to map. Although the most frequent codes were mapped, further mapping and information extraction are needed. NLP methods might be able to recover the free-text information on the diagnosis established during the consultation and the symptoms mentioned.

### Perspective

The next step in the project will involve sharing the ETL process with other health care facilities equipped with Weda software or other software. A qualitative study during the presentation of the dashboard to the health care professionals might improve the prototype dashboard and identify unmet needs for further development. This extension will provide us with an opportunity to conduct multicenter studies and to integrate data from other professions working in primary care (such as nurses, midwives, physiotherapists, and pharmacists).

### Conclusion

We implemented primary care data from a French health care facility into the OMOP CDM format. Data concerning demographics, units, measurements, and primary care consultation steps were already available in the OHDSI vocabularies. Laboratory test results and drug prescription data were mapped with the available vocabulary and structured in the final model. However, the free text in the primary care EHR software complicates the reuse of additional clinical information such as diagnoses, symptoms, clinical reports, and reasons for consultation. A dashboard application provided health care professionals with feedback on their practice.

## Supplementary material

10.2196/49542 Multimedia Appendix 1 Standard concept types for each source table.

10.2196/49542 Multimedia Appendix 2 The distribution of the blood potassium concentration values on the Achilles dashboard on the Atlas server.

10.2196/49542 Multimedia Appendix 3 Results of the data quality assessment dashboard for each feature.

10.2196/49542 Multimedia Appendix 4 List of quality requests and the associated SQL codes.

## Abbreviations

ATC: Anatomical Therapeutic Chemical
CDM: common data model
CIP: *Code Identifiant de Spécialité* (the French national drug code)
EHR: electronic health record
ETL: extract-transform-load
*ICD-10*: *International Classification of Diseases, 10th Revision*
MHC: multidisciplinary health center
NLP: natural language processing
OHDSI: Observational Health Data Sciences and Informatics
OMOP: Observational Medical Outcomes Partnership
PriCaDa: Primary Care Data Warehouse
