# Supplementary material for: Transforming Primary Care Data Into the Observational Medical Outcomes Partnership Common Data Model: Development and Usability Study
Source: JMIR Med Inform. 2024 Aug 13;12:e49542. doi: 10.2196/49542 (PMC11337138; doi:10.2196/49542)
Supplement: Multimedia Appendix 1 [file medinform-v12-e49542-s001.doc]

| Source data | OMOP table | OMOP Source concept id | OMOP Concept type id |
| --- | --- | --- | --- |
|  |  |  |  |
| Medical referrals | NOTE | WW referrals | EHR prescription (32838) |
| Biometric variables | MEASUREMENT  NOTE | WW biometric variables  WW examination report | EHR physical examination (32836)  EHR outpatient note (32834) |
| Death | DEATH | - | EHR (32836) |
| Diagnosis | NOTE | WW diagnosis | EHR pathology report (32835) |
| Drug | DRUG_EXPOSURE | WW drug | EHR prescription (32838) |
| Clinical report by another physician | NOTE | WW clinical report | Health information exchange record (32849) |
| Lab test variables | MEASUREMENT | WW lab test variables | Lab (32856) |
| Medical history | OBSERVATION  NOTE | WW medical history | EHR pathology report (32835) |
| Patient interview | NOTE | WW symptoms | Patient self-report (32865) |
| Additional information | NOTE | WW information | EHR outpatient note (32834) |
| Vaccine prescription | NOTE | WW vaccine names | EHR medication list (32830) |
| Visit | VISIT_OCCURRENCE  NOTE  OBSERVATION_PERIOD | WW outpatient visits  WW reasons  - | EHR (32836)  EHR chief complaint (32822)  Standard algorithm from claims (32881) |

“WW” refers to the EHR software operating at the MHC in Wattrelos.
