# Supplementary figures and images for: Transforming Primary Care Data Into the Observational Medical Outcomes Partnership Common Data Model: Development and Usability Study
Source: JMIR Med Inform. 2024 Aug 13;12:e49542. doi: 10.2196/49542 (PMC11337138; doi:10.2196/49542)

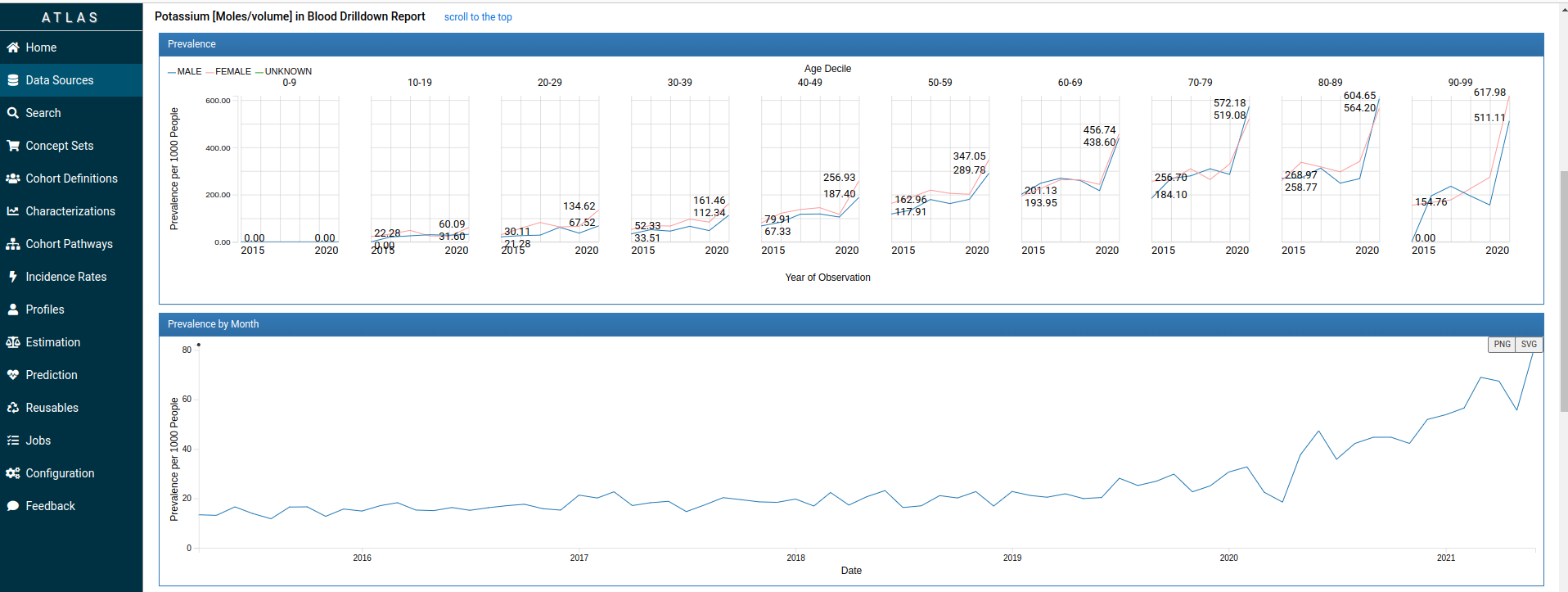

Supplement: Multimedia Appendix 2 [file medinform-v12-e49542-s002.png]

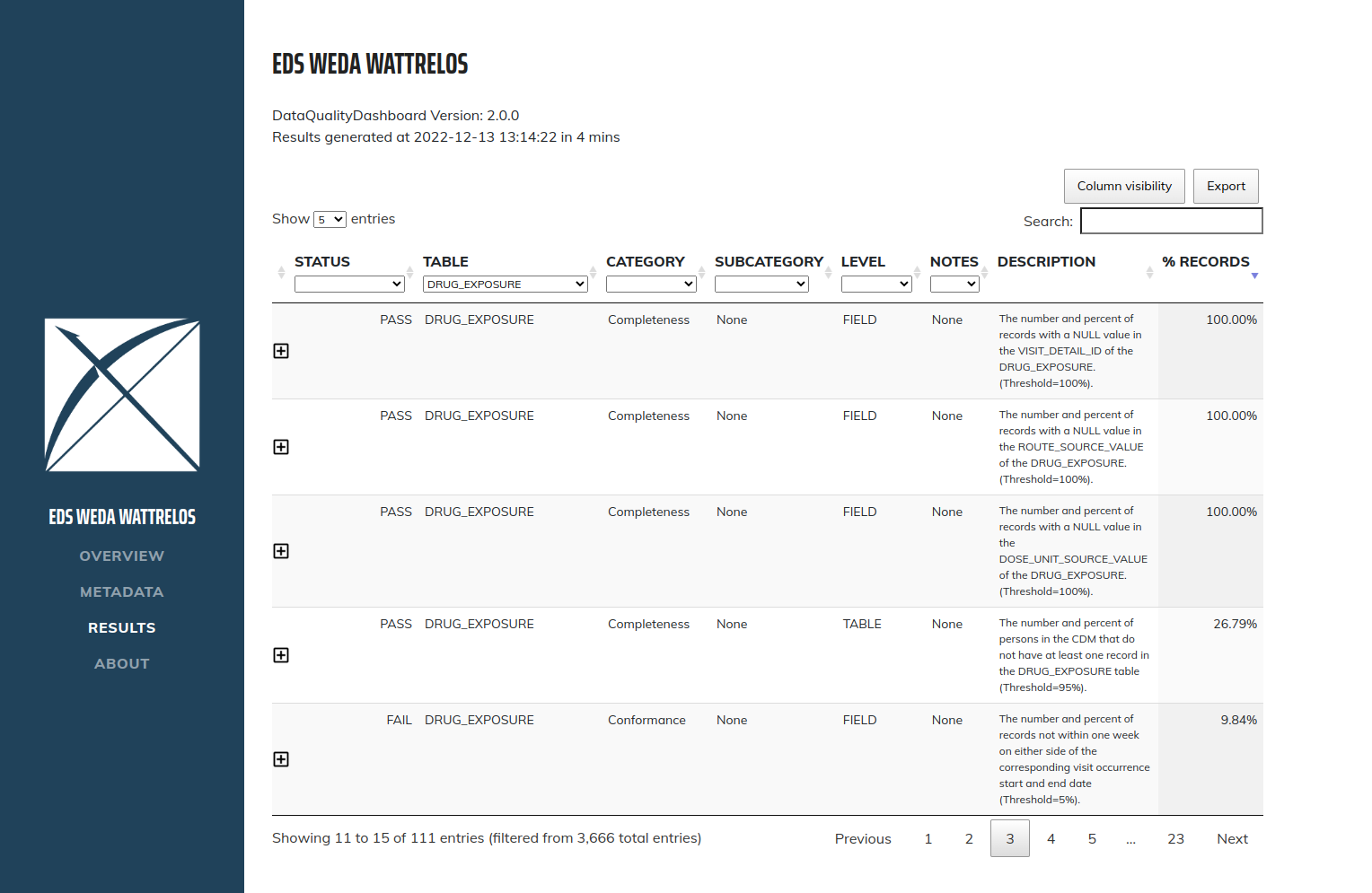

Supplement: Multimedia Appendix 3 [file medinform-v12-e49542-s003.png]
