# Supplementary material for: Transforming Primary Care Data Into the Observational Medical Outcomes Partnership Common Data Model: Development and Usability Study
Source: JMIR Med Inform. 2024 Aug 13;12:e49542. doi: 10.2196/49542 (PMC11337138; doi:10.2196/49542)
Supplement: Multimedia Appendix 4 [file medinform-v12-e49542-s004.doc]

| Request | SQL code | Result | Checking |
| --- | --- | --- | --- |
|  |  |  |  |
| Number of adult patients (aged 18 or over) per family physician seen during the period Jan 1^st^ to Jan 7^th^, 2021. | *SELECT count(distinct(vo.person_id)) as number_patient*  *FROM omop.visit_occurrence vo LEFT JOIN omop.person p ON vo.person_id = p.person_id*  *WHERE vo.visit_start_date >= DATE '01/01/2021'*  *AND vo.visit_end_date <= DATE '07/01/2021' AND (extract(year from vo.visit_start_date) - p.year_of_birth) = 18;* | Dr A: 2 patients  Dr B: 2 patients  Dr C: 2 patients  Dr D: 2 patients | Family physician’s statement after the extraction date (removal of each patient registered with the family physician after the extraction date: verification of the patient records) |
| Number of adult patients (aged 18 or over) seen per family physician in 2020 | *SELECT distinct vo.person_id, p.year_of_birth, p2.provider_name*  *FROM omop.visit_occurrence vo*  *LEFT JOIN omop.person p ON vo.person_id = p.person_id*  *LEFT JOIN omop.provider p2 ON p.provider_id = p2.provider_id*  *WHERE (extract(year from vo.visit_start_date)- p.year_of_birth) >= 18*  *and (extract(year from vo.visit_start_date) - p.year_of_birth) <= 20*  *and vo.visit_start_date >= DATE '01/01/2020'*  *AND vo.visit_end_date <= DATE '31/12/2020'* | Dr E: 8 patients; Dr B: 12 patients; Dr F: 10 patients; Dr C: 4 patients; Dr D: 46 patients; Dr A: 34 patients | Patients who had no consultations in 2020, patients whose file was created after the data extraction date, and patients registered with a GP after the extraction date were removed from the results |
| Number of patients aged 75 and over treated with Zopiclone (ATC: N05CF01) from Jan 1^st^ to Jan 31^st^, 2021 | *SELECT p.gender_concept_id as sex, count(distinct(de.person_id)) as number_patient*  *FROM omop.drug_exposure de*  *LEFT JOIN omop.person p ON de.person_id = p.person_id*  *LEFT JOIN omop.concept_relationship r ON de.drug_concept_id = r.concept_id_1*  *LEFT JOIN omop.concept c ON r.concept_id_2 = c.concept_id*  *WHERE c.concept_code = 'N05CF01'*  *AND c.vocabulary_id = 'ATC'*  *AND de.drug_exposure_start_date >= DATE '01/01/2021'*  *AND de.drug_exposure_start_date <= DATE '31/01/2021'*  *AND (extract(year from current_date) - p.year_of_birth) >= 75*  *GROUP BY p.gender_concept_id;* | 9 female patients and 4 male patients |  |
| Number of patients aged 75 and over treated with Zopiclone (ATC: N05CF01) from Jan 1^st^ to Dec 31^st^, 2021 | *SELECT p.gender_concept_id as sex, count(distinct(de.person_id)) as number_patient*  *FROM omop.drug_exposure de*  *LEFT JOIN omop.person p ON de.person_id = p.person_id*  *LEFT JOIN omop.concept_relationship r ON de.drug_concept_id = r.concept_id_1*  *LEFT JOIN omop.concept c ON r.concept_id_2 = c.concept_id*  *WHERE c.concept_code = 'N05CF01'*  *AND c.vocabulary_id = 'ATC'*  *AND de.drug_exposure_start_date >= DATE '01/01/2020'*  *AND de.drug_exposure_start_date <= DATE '01/01/2021'*  *AND (extract(year from current_date) - p.year_of_birth) >= 75*  *GROUP BY p.gender_concept_id;* | 24 female patients and 9 male patients |  |
| Number of patients aged between 18 and 25 and who had one lab test result between period Jan 1^st^ to Jan 7^th^, 2021 | *SELECT distinct(p.person_id)*  *FROM omop.measurement m*  *LEFT JOIN omop.person p ON m.person_id = p.person_id*  *WHERE m.measurement_source_concept_id = 2000000005*  *AND m.measurement_date >= DATE '01/01/2021'*  *AND m.measurement_date <= DATE '07/01/2021'*  *AND (extract(year from current_date) - p.year_of_birth) >= 18*  *AND (extract(year from current_date) - p.year_of_birth) <= 25* | Patient records: 14 patients.  Data warehouse: 9 patients. | Two lab test results were not filed in the correct place in the software (in the medical reports) and three lab test parameters were not mapped into the data warehouse |
